# The health-related quality of life in patients with Chagas disease: the state of the art

**DOI:** 10.1590/0037-8682-0657-2021

**Published:** 2022-03-14

**Authors:** Igor Lucas Geraldo Izalino de Almeida, Luciano Fonseca Lemos de Oliveira, Pedro Henrique Scheidt Figueiredo, Rafael Dias de Brito Oliveira, Thayrine Rosa Damasceno, Whesley Tanor Silva, Lucas Frois Fernandes de Oliveira, Matheus Ribeiro Ávila, Vanessa Pereira Lima, Ana Thereza Chaves Lages, Mauro Felippe Felix Mediano, Manoel Otávio Costa Rocha, Henrique Silveira Costa

**Affiliations:** 1 Universidade Federal dos Vales do Jequitinhonha e Mucuri, Programa de Pós-Graduação em Reabilitação e Desempenho Funcional, Diamantina, MG, Brasil.; 2 Universidade Federal de Minas Gerais, Escola de Educação Física, Fisioterapia e Terapia Ocupacional, Belo Horizonte, MG, Brasil.; 3 Universidade Federal dos Vales do Jequitinhonha e Mucuri, Departamento de Fisioterapia, Diamantina, MG, Brasil.; 4 Universidade Federal de Minas Gerais, Curso de Pós-graduação em Infectologia e Medicina Tropical, Belo Horizonte, MG, Brasil.; 5 Fundação Oswaldo Cruz, Instituto Nacional de Infectologia Evandro Chagas, Rio de Janeiro, RJ, Brasil.

**Keywords:** Chagas disease, Chagas cardiomyopathy, Quality of life

## Abstract

Chagas disease (CD) is a neglected tropical disease associated with poverty in which patients are surrounded by stigma. These factors can contribute to reducing health-related quality of life (HRQoL). Therefore, a broad discussion of HRQoL in the CD population is required. This study aimed to discuss the main findings of HRQoL in patients with CD, focusing on the association between sociodemographic and lifestyle factors, echocardiographic and functional determinants, and the effect of non-invasive interventions on HRQoL. A literature search of the MEDLINE, Web of Science, CINAHL, Scopus, and LILACS databases was performed with no data or language restrictions. Twenty-two articles were included in this meta-analysis. In general, HRQoL is worse in patients with CD than in healthy individuals, particularly in the presence of cardiovascular and/or gastrointestinal symptoms. Sex, age, functional class, level of physical activity, healthy habits, and medications received could affect HRQoL. Among the echocardiographic and functional determinants, decreased systolic function seems to negatively affect HRQoL. No association with the peak oxygen uptake was observed in the maximal tests. By contrast, well-tolerated field tests with submaximal intensities were associated with HRQoL. Both pharmaceutical care and exercise training have a positive effect on the HRQoL of patients with Chagas cardiomyopathy, and the mental component can be a prognostic marker in this population. In conclusion, assessment of HRQoL can provide important information about the health status of patients with CD, and its use in clinical practice is warranted.

## INTRODUCTION

Chagas disease (CD) is an infection caused by the protozoan *Trypanosoma cruzi* and remains a public health problem in Latin American countries^1^. According to the World Health Organization, the prevalence of CD is estimated at 6 million worldwide, and CD is responsible for 12,000 deaths per year^2^.

In the chronic phase of the disease, patients may present with indeterminate, cardiac, digestive, or mixed forms^3^. In the indeterminate form, the patient remains asymptomatic, with a normal electrocardiogram (ECG) or minor non-specific electrocardiographic abnormalities^4^. Additional investigations using more sophisticated and sensitive complementary methods may reveal changes, such as a higher frequency of exercise-induced ventricular arrhythmias in the exercise test^5^. Gastrointestinal involvement can be detected in the digestive form, marked by the presence of megaesophagus and megacolon^6^. In the cardiac form, patients can progress with symptoms of heart failure such as fatigue and dyspnea, as well as with cardiovascular abnormalities such as malignant arrhythmias and thromboembolism^3^. However, patients can also be asymptomatic despite changes in their cardiac examination results. The cardiac form, denoted Chagas cardiomyopathy (ChC), may present with preserved cardiac function with segmental wall motion impairment until myocardial dilation with mainly left ventricular global systolic dysfunction develops^6^. Dilated ChC is responsible for the higher morbidity and mortality of the disease^3^ ^,^ ^6^ ^,^ ^7^. Finally, the mixed form presents with both cardiac and digestive impairments.

Regardless of the clinical form, interest in assessing the health-related quality of life (HRQoL) of patients with CD has increased in recent decades. Affected individuals are surrounded by stigma, depressive symptoms, social vulnerability, economic and sociodemographic disadvantages, and difficulty in accessing health services^8^ ^-^ ^10^, which contributes to the neglected aspect of the disease. Therefore, the present study aimed to discuss the main findings related to HRQoL of patients with CD. Two previous reviews^11^ ^,^ ^12^ addressed the HRQoL of patients with CD; however, the present study focused on the sociodemographic, lifestyle, echocardiographic, and functional determinants in addition to HRQoL after non-invasive interventions in this population.

## SEARCH METHOD

A narrative review using a structured search strategy was conducted to analyze the main findings regarding HRQoL in patients with CD. Potential studies were identified through a search of the Online Medical Literature Analysis and Retrieval System (MEDLINE), Cumulative Index for Nursing and Allied Health Literature (CINAHL), Web of Science, Scopus, Latin American and Caribbean Health Sciences Literature (LILACS), and Embase databases. The following strategy was used for the PubMed search: ((Chagas disease[Title/Abstract]) OR (Chagas cardiomyopathy[Title/Abstract]) OR (Chagas heart disease[Title/Abstract])) AND ((quality of life[Title/Abstract]) OR (health-related quality of life[Title/Abstract])), which was modified for each database. The search was independently conducted by three authors (ILA, RDBO, and TRD) from June to August 2021.

The inclusion criteria were studies that assessed HRQoL in patients with CD. There were no restrictions on the language or publication year. The exclusion criteria were 1) animal studies, 2) qualitative studies, 3) review studies, and 4) studies that evaluated HRQoL after surgical or invasive procedures. 

**FIGURE 1:**
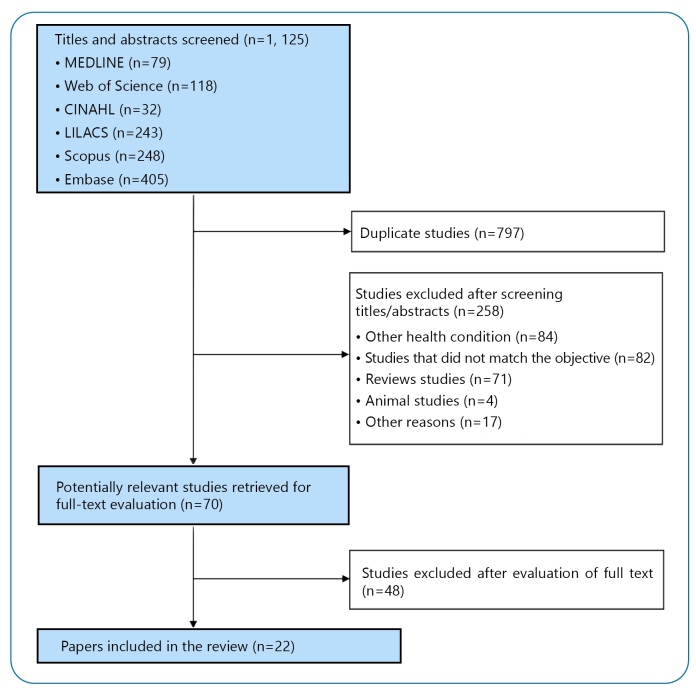
Flow of studies through the review. MEDLINE: Medical Literature Analysis and Retrieval System Online; CINAHL: Cumulative Index to Nursing and Allied Health Literature; LILACS: Latin American & Caribbean Health Sciences Literature.

The original search identified 1,125 articles, of which 797 were duplicates. After reading the titles, abstracts, and objectives, 306 participants were excluded. A total of 22 papers were included in the present review (Figure 1).

Among the included studies, five questionnaires were used: the Short-Form of Health Survey (SF-36)^13^, World Health Organization Quality of Life Questionnaire (WHOQOL-Bref)^14^, Minnesota Living with Heart Failure Questionnaire (MLwHFQ)^15^, Assessment of QUAlity of Life and RELated events (AQUAREL)^16^, and Kansas City Cardiomyopathy Questionnaire^17^.

The SF-36 is a generic questionnaire consisting of 36 items grouped into eight domains (physical functioning, role-physical, bodily pain, general health, vitality, social functioning, role-emotional, and mental health). These domains can be grouped into physical and mental components. The higher the score, the better is the HRQoL. The WHOQOL-Bref is a 24-question questionnaire that includes physical, psychological, social relationship, and environment domains. The higher the score, the better the perception of the HRQoL.

The MLwHFQ is a specific questionnaire for patients with heart failure and consists of 21 questions on patient functionality. The higher the score, the worse the HRQoL. The AQUAREL is a 20-item questionnaire specific to patients with cardiac pacemakers and consists of three domains (chest discomfort, arrhythmia, and exertional dyspnea). Finally, the Kansas City Cardiomyopathy Questionnaire is a self-administered 23-item questionnaire that quantifies physical limitations, symptoms, self-efficacy, social interference, and HRQoL, specifically in patients with cardiomyopathy. The higher the score, the better the perception of HRQoL.

## HRQOL IN PATIENTS WITH CHAGAS DISEASE

Ten studies (Table 1) compared the HRQoL of CD patients with that of healthy individuals, patients with cardiomyopathy from other etiologies, or among the clinical forms of the disease.

**TABLE 1:** HRQoL of patients with Chagas disease (n=10).

Author and year	Sample characteristics	HRQoL questionnaire	Comparison among different clinical forms of Chagas disease/cardiopathies/healthy individuals
Oliveira *et al.* 2008	n=139 cardiac pacemaker patients (40% male) with (n=77) and without (n=31) CD, and individuals with unknown serology (n=31).	AQUAREL	Patients with CD and cardiac pacemakers had worse scores in the chest discomfort (*p=*0.030) and arrhythmia (*p=*0.004) domains compared to non-Chagas patients with cardiac pacemakers.
Gontijo *et al.* 2009	n=70 patients with CD; 68% female, mean age of 53 years, ranging from 27 to 79 years. NYHA and LVEF not reported.	WHOQOL-Bref	Patients with ChC had worse HRQoL in the psychological domains when compared to patients with CD and without heart disease (*p<*0.05). There was no difference in the other domains between the groups.
Oliveira *et al.* 2011	n=146 individuals: 21 without CD [median 46 years (Q1-Q3: 28-71): 62% male; 100% with NYHA I, and 16% with abnormal echocardiography]; 125 with Chagas disease [median 29 years (Q1-Q3: 25-68): 58% male; 82% with NYHA I, and 56% with abnormal echocardiography].	SF-36 and MLwHFQ	The HRQoL of patients with CD was worse in the physical functioning (*p=*0.011) and role-emotional (*p=*0.020) SF-36 domains when compared to non-Chagas disease patients. HRQoL was also worse in the group with CD assessed using the MLwHFQ (*p=*0.028). In patients with CD, the presence of cardiovascular symptoms was associated with poor HRQoL in the physical (OR=4.12) and mental (OR=2.69) component summary of the SF-36. The presence of cardiovascular symptoms was also associated with worse HRQoL in patients with CD when assessed using the MLwHFQ compared to individuals without CD (OR=9.11).
Ozaki *et al.* 2011	n=110 patients with CD (49.09% with ChC: 26.36% with the indeterminate form, 12.73% with the digestive form, and 11.82% with the mixed form); 51% female; mean age of 51 years (ranging from 23 to 82 years). NYHA and LVEF not reported.	WHOQOL-Bref	In the physical domain, the HRQoL of patients in the indeterminate form was significantly better when compared to other clinical forms (*p<*0.0001). Regarding the social relationships domain, there was a significant difference between the indeterminate and cardiac forms (*p<*0.0389). Among those with the chronic phase of CD, those with the digestive form had lower HRQoL scores.
Pelegrino *et al.* 2011	n=43 patients with ChC (62.8% male) and non-Chagas cardiomyopathy (n=59, 57.6% male).	SF-36	Patients with ChC had worse HRQoL in the role-physical (*p=*0.002) and physical functioning (*p=*0.01) SF-36 domains when compared to non-Chagas cardiomyopathy.
Vieira *et al.* 2014	n=16 patients with ChC (53.3±9.2 years, 43.8% female, NYHA I-III, LVEF 34.1±8.0%) and 16 with Chagas disease without cardiopathy (51.9±11.9 years; 50.0% female; NYHA I; LVEF 67.3±5.4%).	MLwHFQ	The group with ChC showed decreased HRQoL in the overall score (p=0.001) and in the physical (*p=*0.031) and role-emotional (*p<*0.001) domains of the MLwHFQ when compared to patients with Chagas disease without cardiopathy.
Ozaki *et al.* 2015	n=202 patients with Chagas disease (66.8% with ChC, 11.4% with the digestive form, and 21.8% with the indeterminate form); 53.96% female; 68.1% aged between 25 and 59 years. NYHA and LVEF not reported.	WHOQOL-Bref	The variables that were associated with worse scores in the physical domain were the digestive and cardiac forms (OR=3.77 and OR=4.42 times more likely, respectively, than the indeterminate form). In the psychological domain, the associated variables were the digestive and cardiac forms (OR=3.33 and OR=2.93 times more likely, respectively, than the indeterminate form). In the social relationships domain, the associated variables were the digestive and cardiac forms (OR=3.63 and OR=2.17 times more likely, respectively, than the indeterminate form.
Shen *et al.* 2017	n=189 patients with dilated ChC (59.6±10.7 years; 66.2% male, NYHA I to III, LVEF 28.5±6.2%); 1101 patients with non-ischemic cardiomyopathy (61.1±12.5 years; 69.0% male; NYHA I to IV; LVEF 27.1±6.3%), 848 patients with ischemic cardiomyopathy (65.8±10.1 years; 78.3% male; NYHA I to IV; LVEF 28.5±6.1%).	Kansas City Cardiomyopathy Questionnaire	Patients with ChC and reduced LVEF have a worse HRQoL than patients with non-ischemic cardiomyopathy (*p=*0.006). There was no significant difference between patients with dilated ChC and patients with ischemic cardiomyopathy (*p=*0.255).
Santos-Filho *et al.* 2018	n=361 patients [indeterminate form (n=97), ChC without heart failure (n=157), ChC with heart failure (n=49), digestive (n=13), cardiodigestive without heart failure (n=38) and cardiodigestive with heart failure (n=7)]; 60.7±10.8 years; 56.3% female; NYHA I to IV; LVEF=57.9±13.9%.	WHOQOL-Bref	In the social relationship domain, the ChC without heart failure was independently associated with worse HRQoL (*p=*0.02). In the environment domain, the cardiodigestive form with heart failure was associated with worse HRQoL (*p=*0.01).
Quintino *et al.* 2020	n=625 patients (65.8% female, 56.7±12.2 years) with non-chagasic cardiomyopathy, ChC without heart failure, and ChC with heart failure.	WHOQOL-Bref	There was no difference in HRQoL among groups in the physical (*p=*0.177), psychological (*p=*0.304), social relations (*p=*0.819) and environment (*p=*0.959) domains.

**Abbreviations: ChC:** Chagas cardiomyopathy; CD: Chagas disease; HRQoL: health-related quality of life; WHOQOL-Bref: World Health Organization Quality of Life Questionnaire, SF-36: Short form of Health Survey; MLwHFQ: Minnesota Living with Heart Failure Questionnaire; AQUAREL: assessment of quality of life and related events; NYHA: New York Heart Association; LVEF: left ventricular ejection fraction; Q1-Q3: interquartile range; OR: odds ratio.

One study^18^ demonstrated that, when compared to healthy individuals, the HRQoL of patients with CD was worse in the SF-36 domains of physical functioning and role-emotional, as well as in the total score of the MLwHFQ. The presence of cardiovascular symptoms in patients with CD was associated with poorer HRQoL in the physical and mental component summaries of the SF-36 as well as in the total score of the MLwHFQ. Therefore, the presence of cardiovascular symptoms seems to significantly contribute to the reduction in HRQoL of patients with CD, a finding that has been verified by other studies.

When comparing patients with CD with and without cardiomyopathy, individuals with cardiac involvement had worse HRQoL in the psychological domain of the WHOQOL-Bref^19^, and in the overall, physical, and role-emotional domains of the MLwHFQ^20^. Thus, the presence of heart disease may worsen the HRQoL of patients with CD, both physically and emotionally. Among the physical aspects, there is a reduction in functional capacity, even in the early stages of heart disease^21^. Regarding emotional aspects, in addition to the stigma surrounding CD, concern about fatalities and fear of sudden cardiac death are aggravating factors^22^.

In a sample stratified among chronic forms of the disease (indeterminate, cardiac, and digestive forms), the presence of cardiovascular or digestive symptoms was associated with worse HRQoL in many domains (physical, psychological, and social relationships) of the WHOQOL-Bref^23^. Thus, it appears that cardiovascular and digestive symptoms are responsible for the poor HRQoL of patients with CD. Esophageal and/or colonic involvement is characterized by dysphagia, odynophagia, esophageal reflux, weight loss, aspiration, cough, regurgitation, and fecaloma^6^. All of these abnormalities contribute to general malaise and social restriction, reducing the HRQoL of patients with the digestive form of CD.

Reduced HRQoL in the cardiac and digestive forms was also found in another study^24^. The authors reported that the cardiac form was associated with worse HRQoL in the WHOQOL-Bref when compared to the indeterminate form; however, the digestive form had the worst scores among the chronic forms. According to Santos-Filho *et al.* ^8^, ChC without heart failure was independently associated with a worse score in the social relationship domain, whereas a mixed form with heart failure was associated with a worse score in the environment domain. More studies are needed to show that the HRQoL of patients with the digestive form is worse than that of patients with ChC; however, so far, it can be stated that both clinical forms have worse scores than patients with the indeterminate form.

Another study^25^ compared the HRQoL of patients with cardiac pacemakers with and without CD using the AQUAREL questionnaire. It was reported that pacemaker patients with CD had worse scores in the chest discomfort and arrhythmia domains than those without CD.

Finally, in a cohort of patients with CD^26^, all of whom had some degree of cardiac impairment, there was no difference in HRQoL assessed by the WHOQOL-Bref in all domains (physical, psychological, social relationships, and environment) between patients with non-Chagas cardiomyopathy, ChC without heart failure, and ChC with heart failure. These results suggest that HRQoL is worse in patients with heart disease, regardless of the etiology or presence of heart failure. In contrast, another study^27^, composed of a population sample with more compromised cardiac function, found that patients with ChC had lower perceived HRQoL in the SF-36 domains of physical functioning and role-physical functioning than those with non-Chagas cardiomyopathy. Similarly, another study^28^ compared HRQoL using the Kansas City Cardiomyopathy Questionnaire in three groups of heart failure: ChC, ischemic, and non-ischemic. The authors demonstrated that patients with ChC had worse HRQoL than those with non-ischemic cardiomyopathy. However, there was no difference between the patients with ChC and those with ischemic cardiomyopathy. Given these conflicting results, more studies are needed to confirm whether Chagas etiology is a determinant of HRQoL in patients with heart disease.

## ASSOCIATION BETWEEN HRQOL AND SOCIODEMOGRAPHIC OR LIFESTYLE FACTORS IN PATIENTS WITH CD

Sociodemographic and lifestyle factors can significantly affect the HRQoL of both healthy individuals and patients with CD. Four studies (Table 2) aimed to verify the association between these factors and HRQoL in patients with CD.

**TABLE 2:** Association between HRQoL and sociodemographic or lifestyle factors (n=4).

Author and year	Sample characteristics	HRQoL questionnaire	Association with sociodemographic or lifestyle variables
Ozaki *et al.* 2011	n=110 patients with Chagas disease (49.09% with ChC, 26.36% with the indeterminate form, 12.73% with the digestive form, and 11.82% with the mixed form); 51% female; mean age of 51 years (ranging from 23 to 82 years). cardiac 49.09%, indeterminate 26.36%, digestive 12.73%, and mixed 11.82%. NYHA=not reported; LVEF=not reported.	WHOQOL-Bref	There was no significant difference when comparing age and marital status with depressive symptom intensity (*p*>0.05). Depressive symptoms and HRQoL, in all domains, were not different between men and women.
Ozaki *et al.* 2015	n=202 patients with Chagas disease (66.8% with ChC, 11.4% with the digestive form and 21.8% with the indeterminate form); 53.96% female, 68.1% aged between 25 and 59 years. NYHA and LVEF not reported.	WHOQOL-Bref	Female sex was associated with the worse scores in the environment domain (*p=*0.033).
Santos-Filho *et al.* 2018	n=361 patients [indeterminate form (n=97), ChC without heart failure (n=157), ChC with heart failure (n=49), digestive (n=13), cardiodigestive without heart failure (n=38) and cardiodigestive with heart failure (n=7)]; 60.7±10.8 years; 56.3% female; NYHA I to IV; LVEF=57.9±13.9%.	WHOQOL-Bref	The variables independently associated with HRQoL were functional class, female sex, clinical presentation of Chagas disease (worse in cardiodigestive with heart failure), sleep duration, schooling, physical activity level, smoking, income per capita, and residents by domicile. The variables associated with the overall HRQoL domain were female sex (*p=*0.03) and worse functional class (p<0.001).
Quintino *et al.* 2020	n=625 patients (65.8% female, 56.7±12.2 years) with non-chagasic cardiomyopathy, Chagas cardiomyopathy without heart failure, and Chagas cardiomyopathy with heart failure.	WHOQOL-Bref	The factors associated with lower HRQoL were age, the use of angiotensin-converting enzyme inhibitors, history of acute myocardial infarction, and no use of angiotensin receptor blockers.

**Abbreviations:** ChC: Chagas cardiomyopathy; HRQoL: health-related quality of life; WHOQOL-Bref: World Health Organization Quality of Life Questionnaire; NYHA: New York Heart Association; LVEF: left ventricular ejection fraction.

In a sample with several chronic forms of the disease (indeterminate, cardiac, and digestive), one study^24^ showed no difference in HRQoL assessed using the WHOQOL-Bref between men and women. However, two other studies^8^ ^,^ ^23^ that included a larger sample with the same chronic forms of the disease and used the same questionnaire showed different results. Ozaki *et al.* ^23^ demonstrated that women had worse scores in the environment domain and were more likely to perceive worse HRQoL than men. Santos-Filho *et al.* ^8^ also demonstrated that women were independently associated with worse HRQoL in the overall score as well as in the physical and psychological domains of the WHOQOL-Bref. Lower HRQoL in women has also been demonstrated in healthy populations^29^ ^,^ ^30^. Compared to men, women have more non-life-threatening diseases as well as a higher prevalence of mental disorders such as depression^29^. In CD, female sex was also associated with depressive symptoms^9^. Thus, we believe that female sex is associated with worse HRQoL in patients with CD.

Santos-Filho *et al.* ^8^ also demonstrated that a worse New York Heart Association (NYHA) functional class, decreased sleep duration, lower schooling, decreased physical activity levels, smoking, decreased income per capita, and residents by domicile were independently associated with poor HRQoL. In another study^26^, increased age, use of angiotensin-converting enzyme inhibitors, history of acute myocardial infarction, and not using angiotensin receptor blockers were also associated with poor HRQoL in patients with CD. 

Age is associated with physical and environmental domains, and functional impairment is common with increasing age^31^, which negatively affects HRQoL. A history of a previous acute myocardial infarction was associated with worse scores in the social relationship domain, which may be explained by a lower perception of emotional support and greater fear of social interactions after a myocardial infarction^32^. Regarding the medications received, the use of angiotensin-converting enzyme inhibitors was associated with worse HRQoL in the physical domain. A common adverse effect of angiotensin-converting enzyme inhibitor is cough^33^, which may impact the physical domain of HRQoL, especially at high doses. In contrast, the use of angiotensin receptor blockers was associated with a better HRQoL in patients with CD. Angiotensin receptor blockers have a low incidence of adverse effects and are associated with better HRQoL than other therapies for patients with arterial hypertension and/or heart failure^34^.

## ASSOCIATION BETWEEN HRQOL AND FUNCTIONAL VARIABLES, ECHOCARDIOGRAPHIC PARAMETERS, OR DISABILITIES

Seven studies verified the association between HRQoL and functional capacity, disability, and/or echocardiography findings (Table 3).

**TABLE 3:** Association between HRQoL and echocardiographic, functional, and disabilities parameters (n=6).

Author and year	Sample characteristics	HRQoL questionnaire	Association with functional and echocardiographic parameters
Dourado *et al.* 2006	n=61 patients with ChC and heart failure, (mean age: 50±14 years; 71% male; NYHA I to IV; LVEF=33.5±12%.	MLwHFQ	The 6MWT distance was correlated with the MLwHF total score (r=-0.4; *p<*0.001), physical dimension (r=-0.42; *p=*0.001) and emotional dimension (r=-0.33; *p=*0.008) domains.
Ritt *et al.* 2013	n=55 patients with ChC and LVEF<45% (NYHA II to IV, LVEF=27.6±6.6%).	MLwHFQ	The HRQoL was correlated with VO2peak (*r=*−0.301, *p=*0.02), 6MWT distance (*r=*−0.375, *p=*0.007), and LVEF (*r=*−0.282, *p=*0.03). These variables explained 30% of the variation in the MLwHFQ.
Souza *et al.* 2013	n=21 patients with Chagas disease after stroke (50.2±13.9 years; 57% male; NYHA and LVEF not reported).	WHOQOL-Bref	There was no correlation between disability, assessed by Modified Rankin Stroke Scale, with any of the WHOQOL-Bref domains [physical (*r=*-0.207; *p=*0.410), psychological (*r=*0.017; *p=*0.946), environment (*r=*0.511; *p=*0.830), and social relationship (*r=*0.229; *p=*0.360)].
Costa *et al.* 2014	n=35 patients with ChC; mean age: 47.1±8.2 years; 65.7% male; NYHA I to III; median LVEF=59% (interquartile range from 41 to 46%).	SF-36 and MLHFQ	The ISWT distance was correlated with the MLwHFQ total score (r=-0.460; *p=*0.06), and some SF-36 domains [physical functioning (*r=*0.435; *p=*0.009), role-physical functioning (*r=*0.447; *p=*0.008), and mental health (*r=*0.430; *p=*0.011)]. The VO2peak was correlated only with the physical functioning domain of the SF-36 (*r=*0.383; *p=*0.025). There was no correlation between VO2peak and MLwHFQ score (r=-0.337; *p=*0.055).
Chambela *et al.* 2017	n=53 patients with ChC; mean age: 60±12 years; 48.8% female; NYHA I to III; LVEF=35.1±11.1%.	SF-36 and MLHFQ	There was a significant correlation between the 6MWT distance and MLwHFQ total score (r=-0.54; *p=*0.002) and some SF-36, domains [physical functioning (r=0.46; *p=*0.008), role-physical functioning (*r=*0.37; *p=*0.04), and bodily pain (*r=*0.43; *p=*0.014)].
Ávila *et al.* 2021	n=75 patients with ChC; mean age: 49 years (95% CI: 47 to 51); 46% male; NYHA I to III; LVEF=44% (95% CI: 41 to 48%).	SF-36	Patients with systolic dysfunction have a worse HRQoL in the physical functioning (p<0.001), role-physical functioning (*p=*0.041), and general health perception (*p=*0.013) domains when compared to those who have preserved systolic function. The best cut-off points in identifying patients with systolic dysfunction were scores ≤46 and ≤54 in the physical and mental components of the SF-36, respectively.

**Abbreviations:** ChC: Chagas cardiomyopathy; HRQoL: health-related quality of life; WHOQOL-Bref: World Health Organization Quality of Life Questionnaire; NYHA: New York Heart Association; LVEF: left ventricular ejection fraction; SF-36: Short-Form Health Survey; MLwHFQ: Minnesota Living with Heart Failure Questionnaire; VO2peak: peak oxygen uptake; 6MWT: six-minute walk test; ISWT: incremental shuttle walk test.

Systolic dysfunction, assessed by left ventricular ejection fraction (LVEF), is a well-established prognostic marker in the CD population^35^ ^-^ ^37^, and two studies^38^ ^,^ ^39^ have verified the association between HRQoL and cardiac function. One study in patients with ChC and heart failure (n=55, LVEF <45%) demonstrated a weak but significant correlation between HRQoL, as assessed by the MLwHFQ and LVEF. According to the authors, the lower the LVEF, the worse the HRQoL of the patient. Ávila *et al.* ^39^ showed an association between HRQoL and systolic dysfunction in patients with ChC. The authors stratified the sample into groups according to systolic dysfunction and preserved cardiac function. The groups with systolic dysfunction had worse QoL in the domains of physical functioning, physical role functioning, and general health perception. In addition, the accuracy of the SF-36 in identifying patients with systolic dysfunction was demonstrated. The physical component of the SF-36 showed good efficacy in identifying these patients. A score of <46 points was the optimal cutoff point for diagnostic accuracy, with a positive predictive value of 91%. Therefore, the physical component of SF-36 can be used as a risk stratification and screening tool for patients with ChC, especially when echocardiography is scarcely available.

Functional capacity, assessed by both peak oxygen uptake (VO2peak) and field tests, has clinical and prognostic importance in patients with ChC^40^. Two studies included in this review verified the association between VO2peak and HRQoL, assessed using the MLwHFQ and SF-36. One study^41^ with a sample of patients with both systolic dysfunction and preserved cardiac function found no correlation between VO2peak and MLwHFQ scores. The authors also used the SF-36 questionnaire and only the physical functioning domain showed a significant correlation. Another study^38^ in patients with ChC and heart failure found a weak but significant correlation between VO2peak and MLwHFQ score. These findings suggest that VO2peak and maximal functional capacity may not reflect HRQoL in patients with ChC. We hypothesized that HRQoL is more strongly associated with daily activities, usually performed at a submaximal level. Therefore, field tests can be useful tools for investigating patients’ perceptions of their health.

Two field tests have already been applied in patients with ChC for functional assessment: the six-minute walk test (6MWT)^20^ ^,^ ^42^ ^-^ ^45^ and incremental shuttle walk test (ISWT)^46^ ^-^ ^48^. The 6MWT is a field test that evaluates functional capacity by the distance covered in six minutes^49^. In patients with preserved cardiac function, the 6MWT distance was not correlated with the presence of depressive symptoms^9^. In patients with ChC and systolic dysfunction, the 6MWT distance was correlated with the MLwHFQ total score^38^ ^,^ ^50^ ^,^ ^51^ and with the SF-36 domains of physical functioning, role-physical functioning, and bodily pain^50^. It has also been shown that, among functional variables, the 6MWT distance was the only determinant of HRQoL^38^. A 10-m increase in the 6MWT distance is associated with a reduction of 0.7 points in the MLwHFQ score.

The ISWT is a symptom-limited field test with progressive loads and 12 levels of intensity, where the functional capacity is evaluated by the walked distance^52^. In patients with ChC, only one study^41^ verified the association between ISWT distance and HRQoL using both the SF-36 and MLwHFQ. The authors demonstrated that ISWT distance was correlated with MLHFQ total score and the physical functioning, role-physical functioning, and mental health domains of the SF-36. The results regarding the association between HRQoL and the field tests corroborate our hypothesis that submaximal tests are more representative of HRQoL than maximal tests.

Regarding disability, one study^53^ verified the association between HRQoL using the WHOQOL-BREF and the degree of disability using the Modified Rankin Stroke Scale in patients with CD after stroke. It has been shown that cerebrovascular events are frequent in patients with CD, and these may be the first clinical manifestation of the disease^54^. However, the authors found no association between disability and the WHOQOL-Bref domains. Disability was associated with functional performance, whereas HRQoL was associated with depressive symptoms.

## HRQOL ASSESSMENT IN LONGITUDINAL STUDIES

Recent studies^55^ ^,^ ^56^ have highlighted the importance of assessing HRQoL in clinical trials as an effective tool to detect patient-reported changes. Thus, HRQoL has the potential to identify improvements in health from proposed interventions and can be used as a valuable prognostic marker^56^.

Six longitudinal studies^57^ ^-^ ^62^ assessed the HRQoL of patients with ChC (Table 4). Five of them^57^ ^-^ ^60^ ^,^ ^62^ evaluated the effects of physical interventions or drug therapies on HRQoL, and one observational study^61^ verified the prognostic value of HRQoL in patients with ChC.

**TABLE 4:** The use of HRQoL assessment questionnaires in longitudinal studies (n=6).

Author and year	Sample characteristics	HRQoL questionnaire	Follow-up	Results
Botoni *et al.* 2007	n=39 patients with ChC; 47.8±10.4 years; 71% male; NYHA I to III; FEVE 43.2±14.5%. Groups were stratified into control group (received enalapril and spironolactone, n=20) and intervention group (received carvedilol after enalapril and spironolactone, n=19).	SF-36	Drug therapy (use of carvedilol after renin-angiotensin system inhibition)	Optimization of RAS inhibition was associated with improvements in the SF-36 total score (*p=*0.0003), including in the physical functioning (*p=*0.046), role-physical functioning (*p=*0.002), bodily pain (*p=*0.021), general health perceptions (*p<*0.001), and mental health (*p=*0.033) domains. The subsequent use of carvedilol did not improve any SF-36 domain.
Lima *et al.* 2010	n=40 patients with dilated ChC, stratified in an inactive control group (n=19, 36% female, NYHA I to II, LVEF 37.0±7.6%) and an exercise training group (n=21, 48% female, NYHA I to II, LVEF=35.7±8.1%).	SF-36	Exercise training (12 weeks, 3 times per week, at moderate intensity)	Exercise training improved the intergroup HRQoL in the vitality (*p=*0.013), role-emotional (*p=*0.012), and mental health (*p=*0.031) domains of the SF-36.
Mediano *et al.* 2016	n=12 patients with ChC and heart failure (single group, 56.1±13.8 years, 75% female; NYHA I to III; LVEF=31.9±7.7%).	MLwHFQ	Exercise training (8 months, 3 times per week, 60 minutes per session, at moderate intensity)	Patients with right ventricular dysfunction at baseline exhibited improvements in MLwHFQ total score (*p=*0.009). Improvements in MLwHFQ were not observed among those without right ventricular dysfunction.
Mediano *et al.* 2017	n=12 patients with ChC and heart failure (single group, 56.1±13.8 years, 75% female; NYHA I to III; LVEF=31.9±7.7%).	SF-36	Exercise training (8 months, 3 times per week, 60 minutes per session, at moderate intensity)	Exercise training led to improvements in the physical functioning (*p=*0.003), role-physical functioning (*p=*0.03), and bodily pain (*p=*0.02) SF-36 domains, as well as in the physical component summary (*p=*0.001) domain. P­­­atients with right ventricular dysfunction demonstrated significant improvements in the physical functioning (*p=*0.001), bodily pain (*p=*0.02), and vitality (*p=*0.03) SF-36 domains, and in the physical component summary (*p=*0.001). Patients with preserved right ventricular function showed significant improvements only in the physical component summary (*p=*0.002).
Costa *et al.* 2018	n=75 patients with ChC (with and without systolic dysfunction), 48.4±8.0 years; 39% female, median LVEF=41.0% (Q1-Q3 35.0-53.5); NYHA I to III.	SF-36	Observational (six years of follow-up)	After the follow-up period, the general health (*p=*0.047) and social functioning (*p=*0.026) SF-36 domains, as well as the mental component summary (*p=*0.043), were significantly different between the groups with and without adverse cardiovascular events. In the final multivariate Cox regression model, LVEF (HR 0.94, 95% CI from 0.90 to 0.98, *p=*0.007) and the mental component summary of the SF-36 (HR 0.98, 95% CI from 0.94 to 1.00, *p=*0.047). remained as independent predictors of adverse cardiovascular outcome in patients with ChC.
Chambela *et a*l. 2020	n=81 patients with ChC and heart failure, 61±11 years, 52% female, NYHA I to III, LVEF=36.0±9.9%. Groups were stratified into standard care (n=41) and pharmaceutical care (n=40).	SF-36 and MLwHFQ	Drug therapy (one year of follow-up)	When compared with the standard care group, patients under drug therapy, after one year, showed improvements in the physical functioning (*p<*0.001), role-physical functioning (*p=*0.01), general health perceptions (*p<*0.001), vitality (*p=*0.003), social functioning (*p=*0.002), and mental health (*p=*0.006) domains. Improvement in HRQoL, as assessed by the MLwHFQ, was also higher in those under drug therapy compared to those under standard care after one year (*p<*0.001).

**Abbreviations:** ChC: Chagas cardiomyopathy; HRQoL: health-related quality of life; WHOQOL-Bref: World Health Organization Quality of Life Questionnaire, SF-36: Short form of Health Survey; MLwHFQ: Minnesota Living with Heart Failure Questionnaire; NYHA: New York Heart Association; LVEF: left ventricular ejection fraction; 95% CI: 95% confidence interval; HR: hazard ratio.

The reassessment of HRQoL after drug therapy was verified in two studies. Chambela *et al.* ^57^ found that the group of patients with ChC and heart failure who received optimized drug therapy (n=40) showed a significant improvement in many domains of the SF-36 and in the total score of the MLwHFQ compared to the group receiving standard care (n=41). Therefore, the results suggest that both the SF-36 and MLwHFQ may be sensitive in identifying improvements in the health perception of patients with ChC and heart failure after drug therapy. Another study^62^ that verified HRQoL after pharmacological treatments was carried out in two stages. First, all patients with ChC (n=39) were administered enalapril and spironolactone. Subsequently, there was a significant improvement in their SF-36 total scores, including in the physical functioning, role-physical functioning, bodily pain, general health perceptions, and mental health domains. Second, patients in the experimental group (n=19) received carvedilol, while those in the control group (n=20) received a placebo. There was no difference in any of the SF-36 domains between the groups after treatment with carvedilol. In addition, no improvement in hemodynamic, echocardiographic, or circulating chemokine parameters was observed.

The effects of exercise training on HRQoL were demonstrated in three studies^58^ ^-^ ^60^, all of which included patients with systolic dysfunction. One study^60^, which applied a three-month moderate-intensity intervention, showed improvement in the vitality, role-emotional, and mental health domains of the SF-36 in the exercise group when compared to the inactive group. The improvements in the mental and emotional factors were greater than in the physical factors, despite the improvement in the functional capacity. The authors highlighted that interpersonal contact during the exercise program was important for increasing well-being and improving the psychosocial aspects. Another study^59^ showed improvements in the domains of physical functioning, role-physical functioning, and bodily pain, as well as in the physical component summary, after 8 months of an exercise intervention. The study consisted of a single-arm intervention that included 12 patients with ChC and heart failure. In a reassessment analysis^58^ including the same patients, the authors demonstrated an improvement in the total MLwHFQ score occurred only in patients with right ventricular dysfunction. The beneficial changes in HRQoL also accompanied the clinical changes in these patients, and individuals with the greatest severity of cardiac impairment obtained the most substantial benefits in cardiac hemodynamics, respiratory strength, and HRQoL.

Finally, in a study of 75 patients with ChC, Costa *et al.* ^61^ verified the prognostic value of HRQoL in predicting adverse cerebrovascular events. After six years of follow-up, the mental component of the SF-36 together with LVEF remained an independent predictor of adverse events. The physical component did not show significant prognostic value; however, the sample was predominantly composed of patients with preserved functional class, and studies with patients with functional impairment should be conducted. Therefore, the findings suggest that HRQoL, especially the mental aspects, should be used in clinical follow-ups, since the patient can be aware of the progression of the disease.

## FINAL CONSIDERATIONS

The results of the included studies suggest that 1) HRQoL is worse in patients with CD than in healthy individuals; 2) the presence of cardiovascular and gastrointestinal symptoms are responsible for worse HRQoL scores in terms of both physical and mental aspects; 3) the HRQoL in patients with ChC compared to those with other heart diseases is still poorly understood; 4) female sex is associated with worse HRQoL; 5) other factors, including age, functional class, level of physical activity, healthy habits, and medications, can affect the HRQoL of patients; 6) HRQoL is related to systolic function; 7) functional capacity assessed by VO2peak may not reflect the HRQoL in ChC; 8) field tests may be associated with HRQoL; 9) drug therapy, in general, has a positive effect on the HRQoL of patients with ChC; 10) exercise training can also positively impact HRQoL in both physical and emotional aspects; and 11) HRQoL, especially the mental component, can be a prognostic marker in patients with ChC.
